# The challenges and considerations of community-based preparedness at the onset of COVID-19 outbreak in Iran, 2020

**DOI:** 10.1017/S0950268820000783

**Published:** 2020-04-03

**Authors:** Rahmatollah Moradzadeh

**Affiliations:** Department of Epidemiology, School of Health, Arak University of Medical Sciences, Arak, Iran

**Keywords:** COVID-19, epidemics, epidemiology, Iran, outbreak

## Abstract

COVID-19 as an emerging disease has spread to 183 countries and territories worldwide as of 20 March 2020. The first COVID-19 case (i.e. the index case) in Iran was observed in the city of Qom on 19 February 2020. One of the cities of Markazi Province is Delijan, which shares a border with Qom. Consequently, COVID-19 has quickly spread in this city because a large population commutes daily between the two cities. This study aimed to report the challenges and considerations of community-based preparedness at the onset of COVID-19 outbreak in a city of Iran in 2020.

## Introduction

COVID-19 as an emerging disease has spread to 183 countries and territories worldwide as of 20 March 2020. The total number of COVID-19 cases has been 266 073 across the world [1] and 19 644 cases in Iran [1–4] at the time of writing this report (20 March 2020). Of all these patients, 11 184 cases have died worldwide [1, 3] and 1433 cases have died in Iran [1, 4]. The first COVID-19 case (i.e. the index case) in Iran was observed in the city of Qom on 19 February 2020. COVID-19 has started to spread rapidly in Qom and other neighbouring provinces, including Markazi Province, ever since. This study aimed to report the challenges and considerations of community-based preparedness at the onset of COVID-19 outbreak in a city of Iran in 2020.

## Description of the situation in Delijan

One of the cities of Markazi Province is Delijan which shares a border with Qom. Delijan is located in the Iranian section of the International North−South Transport Corridor and considered as the main route connecting the northern half of the country to the southern counterpart. This city is located 94 km from Qom and its population is 55 000 people. A total of 353 COVID-19 cases has been detected in Qom thus far. The first COVID-19 case in Delijan was from a taxi driver who had visited Qom a few days before his diagnosis. Consequently, COVID-19 has quickly spread in this city because a large population commutes daily between the two cities. This prompted the epidemiological team of the Arak University of Medical Sciences to visit the city.

## Response, case definition used and case finding

The visit was conducted in Delijan on 2 March 2020, and we observed that 20 hospitalised people had COVID-19 (the laboratory results were reported after a 48 h delay, that is, the reported cases belonged to 29 February 2020 and earlier), six people were dead and 54 people were probable cases. The definitions of probable and confirmed cases is based on the World Health Organization guidelines [5]. Our field observation revealed that the streets of the city were sparsely populated and few shops were open; yet, many supermarkets were open. We also received a report on the implemented medical measures from the city's health centre.

## Results and discussion

These are some considerations that may arise in this city and any affected cities around the world in the early stages of the epidemic and some problems which should be preplanned to take necessary measures. COVID-19 is transferred by a propagated epidemic [6] and the reproductive rate is 2.28 (2.06–2.52) [7, 8].

Among 20 COVID-19 cases, three villages near Delijan had positive cases, but other COVID-19 cases (*n* = 17) were observed in the city. Four of the COVID-19 patients were the medical staff of hospitals. Since only those cases with severe symptoms undergo confirmatory diagnostic tests and given that the spreading pattern in Iran is similar to that of China (the percentage of cases with severe COVID-19 were 13.8% and 6.1% critical conditions in China) [9], we expect that about 20% of the patients are severe and critical cases in Iran. Thus, given the fact that 20 patients are suffering from severe, critical COVID-19 symptoms, it can be estimated that there are at least 80 patients in Delijan with mild-to-moderate symptoms who have gone undetected and hence able to easily transfer COVID-19 to others.

**Fig. 1.**
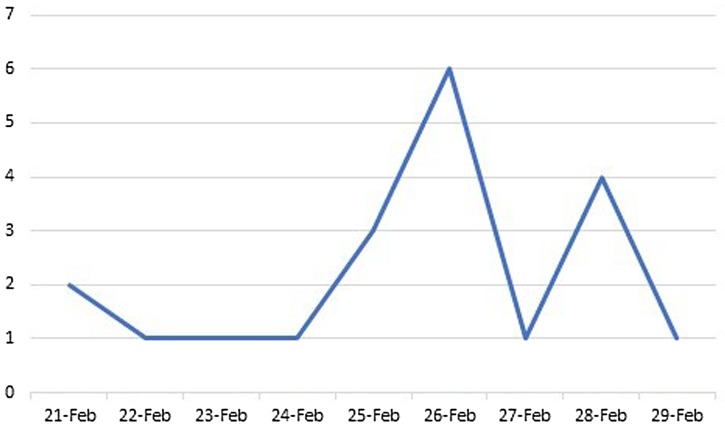
Epidemic curve of confirmed cases of COVID-19 in Delijan from 21 February 2020 to 29 February 2020.

The epidemic curve of COVID-19 is shown in Figure 1. The detected probable cases are not much higher than the confirmed cases showing that the process of case finding has not been done on time or that many people have been turned into the asymptomatic types of COVID-19. The proportion of asymptomatic patients may be high. Nevertheless, we can evaluate the situation more thoroughly in the coming days and later phases of the epidemic.

An important point to consider is that the transmission route of 60% (*n* = 12) of the cases has been unidentified. Four cases have been reported among health workers but only two patients mentioned transmission routes; others had no meaningful relationship with other cases with the only exception of one case who had visited Qom earlier.

It is essential to provide disease mapping separately for confirmed, probable and suspected cases. Likewise, we suggest that the clustering of cases and hot spots should be identified in this region so that case finding and contact tracing are conducted with heightened sensitivity.

On the local scale, we recommend that the trend of COVID-19 should be observed and followed by scientific methods [10]. It is also necessary to pay close attention to family and out-of-family contact tracing according to the latest definitions of contact [5].

A minority group of immigrant Afghans live in an area in the city. Some of them have residency certificates, but others do not. No COVID-19 cases have been identified among them so far maybe because they are unwilling to disclose their health conditions. Although the paraclinical and medical costs of COVID-19 patients are free, these people might be afraid of being quarantined or being deported to their country, and hence maybe hiding their true health conditions. Accordingly, necessary measures have been recommended to build trust among them. These trust building measures were conducted by the head of health system and the governor of Delijan. Local elders, who are trusted by immigrant Afghans, are also included.

People are generally scared that apart from the epidemic of COVID-19, they are also facing an infodemic [11], we have recommended that video clips should be recorded and uploaded on social networks in which the governor, the religious leader and the local head of the health system provide people with correct up-to-date information. There are several factories in the suburbs where workers are raising objection as to why they have not closed down. Thus, health centres are under duress to order the closing down of such factories and production lines.

It is of high importance to register the contact cases of under one meter [12]. Unfortunately, human resources and diagnostic equipment are not sufficient for the timely identification of COVID-19. This has made health centres and hospitals prescribe confirmatory diagnostic tests only for those patients who show severe COVID-19 symptoms. The identification of the secondary and tertiary contacts should be prioritised in control programmes. Those contacts will be encouraged to stay at home, as they will be notified that they are contacts of confirmed cases. There is an official advice on self-isolation with or without symptoms. Household members of confirmed cases are recommended to stay at home and without close contact with other healthy household members, and they are regularly followed up by health care workers. Moreover, voluntary home quarantine (self-isolation) is recommended for an asymptomatic person, when they have a high risk of exposure to the virus that causes COVID-19 (i.e. through close contact with a symptomatic person or their body fluids). The population is asked to self-isolate in the home setting to avoid contact with others in order to prevent transmission of COVID-19. The current situation of the personal protective equipment (PPE) is insufficient. There is inadequate access to PPE even for the health care workers. The health care workers without symptoms did not undergo tests regularly, and it is expected that more cases may surface among them.

## Conclusion

These considerations were provided by the epidemiological team of the Arak University of Medical Sciences in order to providing scientific solutions to the provincial authorities to find barriers and problems for local staff to control the epidemic in Delijan. These considerations can be generalised to the region, the country and outside Iran.

Consequently, health strategies and programmes need to take into account the justification and appropriate education of health workers to make them ready to deal with epidemics.
